# Comprehensive identification of sequence types belonging to Acinetobacter baumannii clonal complexes

**DOI:** 10.1099/mgen.0.001772

**Published:** 2026-07-01

**Authors:** Ruth M. Hall, Christopher J. Harmer

**Affiliations:** 1 School of Life and Environmental Sciences, The University of Sydney, Sydney, NSW, Australia; 2 The University of Sydney Infectious Diseases Institute (Sydney ID), The University of Sydney, Sydney, NSW, Australia

**Keywords:** *Acinetobacter baumannii*, genomics, MLST, sequence type

## Abstract

*Acinetobacter baumannii* is a major nosocomial pathogen, with multiply antibiotic-resistant (MAR) isolates primarily belonging to two globally disseminated clonal complexes, Global Clone 1 (GC1) and Global Clone 2 (GC2). *A. baumannii* has two MLST schemes: the Pasteur and the Oxford scheme. However, the Pasteur scheme identifies clones more simply. Under the Pasteur MLST scheme, these clones centre on sequence types 1 (ST1) and 2 (ST2), respectively, but both encompass related single-locus variants (SLVs) and double-locus variants (DLVs) and even some triple-locus variants (TLVs). Despite their clinical importance, no systematic and up-to-date catalogue of sequence types (STs) belonging to GC1 or GC2 was available.

A workflow was developed using a custom Python workflow to identify SLVs and DLVs of founder STs. This identified 63 STs associated with ST1 and 163 associated with ST2. The impact of accounting for these additional ST was evaluated by analysing 41,951 publicly available *A. baumannii* genome assemblies from GenBank. Inclusion of SLVs and DLVs increased the number of GC1 genomes significantly from 1,083 ST1 to 1,428 genomes total, but GC2 only from 26,962 to 28,002 genomes. Relatively few SLVs or DLVs found in PubMLST were represented among genome assemblies (22 out of 64 for ST1 and 65 out of 164 for ST2), suggesting incomplete genome coverage or erroneous MLST profiles. Other successful clones, including ST25, ST79, ST85 and ST499, each showed diversification. This framework provides a method for consistent clone definition across clinically important *A. baumannii* clonal complexes and can be applied more broadly to other important MAR bacteria such as *Klebsiella pneumoniae*.

**Impact Statement**

Accurate clone assignment is fundamental to understanding the epidemiology and evolution of *Acinetobacter baumannii*. Although Global Clone 1 (GC1) and Global Clone 2 (GC2) are widely recognized as the dominant global lineages, most studies refer only to the founder sequence types (STs) and often overlook the broader sets of related STs that form their true clonal complexes. This practice can lead to inconsistent nomenclature, incomplete surveillance and difficulties comparing findings across studies.

This work provides the first systematic and reproducible catalogue of all Pasteur MLST STs belonging to GC1 and GC2, based on single-locus and double-locus variant analysis. Using a custom Python workflow applied to the entire PubMLST database and more than 41,000 publicly available genomes, we show that both GC1 and GC2 encompass far more STs than are typically recognized. Incorporating these related STs increases the number of genomes assigned to each clone by several hundred, revealing a considerable amount of diversity that is not otherwise captured.

The same approach was extended to other major epidemic complexes, including ST25, ST79, ST78, ST85 and ST499, demonstrating applicability beyond the global clones. This framework strengthens the accuracy and consistency of lineage assignment and provides a robust foundation for future comparative genomics and molecular epidemiology of *A. baumannii*.

## Data Availability

The Python script and data tables utilized in this analysis are publicly available in GitHub (https://github.com/charmer-git/ST). All *Acinetobacter baumannii* MLST allele sequences and sequence type definitions from the Pasteur scheme are publicly available from the PubMLST database (https://pubmlst.org/organisms/acinetobacter-baumannii). All genomes examined in this study were downloaded from the NCBI FTP site (https://ftp.ncbi.nlm.nih.gov/).

## Introduction

*Acinetobacter baumannii* has emerged as one of the most important nosocomial pathogens worldwide. Its population structure comprises multiple well-defined clonal complexes (CCs), each centred on a founder sequence type (ST) and encompassing related variants that have diversified through mutation and recombination. Among these CCs, Global Clone 1 [GC1 (CC1)] and Global Clone 2 [GC2 (CC2)] are recognized as true global clones, having disseminated widely across multiple continents over the past three decades [1,3]. Together, GC1 and GC2 account for the majority of *A. baumannii* infections associated with healthcare settings [3,7] and dominate the publicly available genome sequence data [8,10]. Their global success distinguishes them from other epidemic CCs. Several other complexes, such as those founded by ST25 [11,12], ST78 [13,14], ST79 [15] and ST499 [16], are also epidemiologically successful but tend to be more regionally constrained or associated with specific clinical settings.

**Fig. 1.**
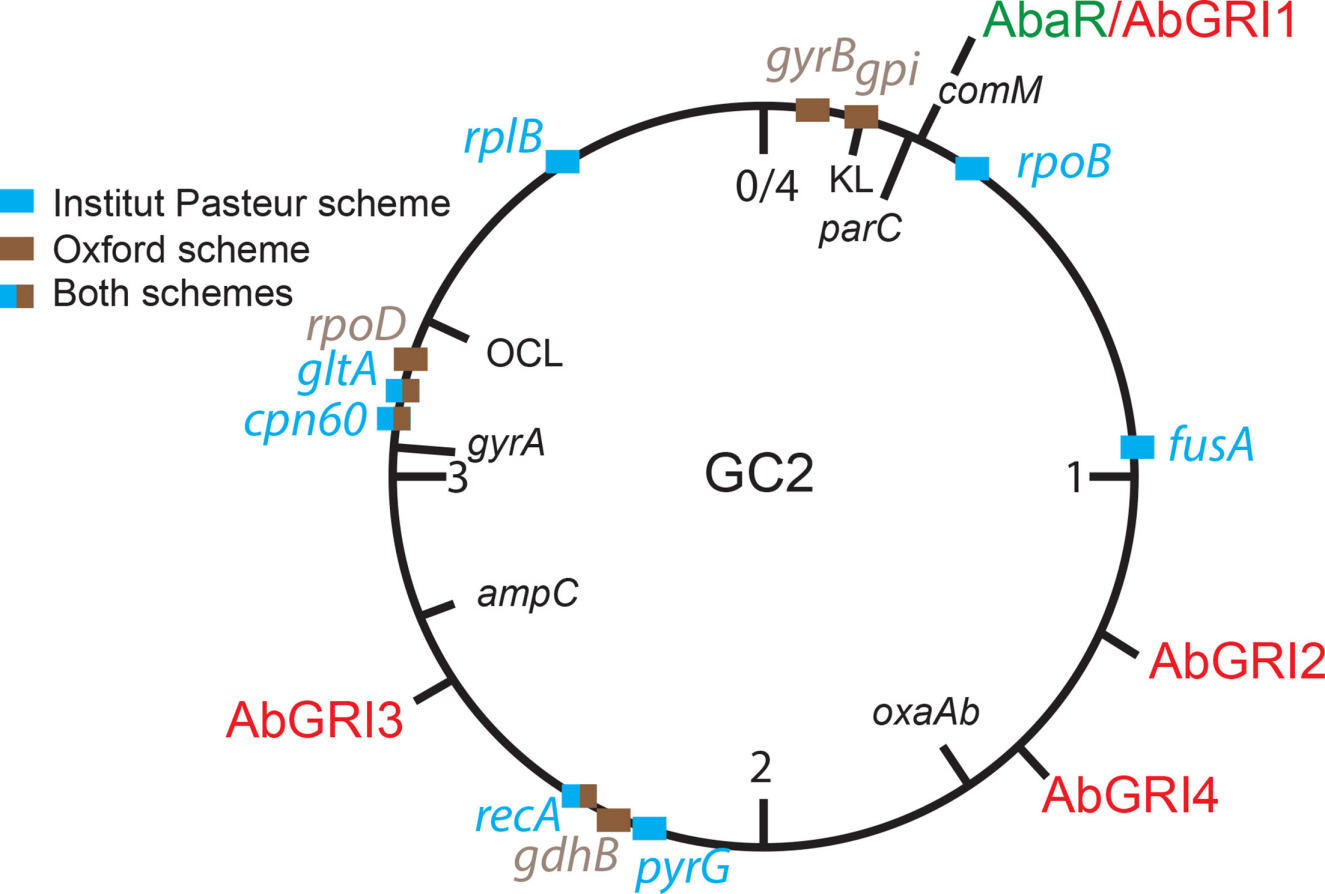
Circular schematic of the *A. baumannii* chromosome showing the position of genes used in the Institut Pasteur and the Oxford MLST schemes. The locations of the capsule biosynthesis (KL) and outer core (OCL) loci and other important genes (*comM*, *ampC*, *oxaAb*, *parC* and *gyrA*) are also marked. The position of the characteristic GC1 (AbaR) and GC2 (AbGRI1, AbGRI2, AbGRI3 and AbGRI4) antibiotic resistance islands is also marked.

Molecular typing approaches, including MLST, have been instrumental in defining the population structure of *A. baumannii*. Two MLST schemes are in use for *A. baumannii*: the Institut Pasteur [17] and Oxford schemes [18]. The Pasteur scheme is generally considered more robust for population-level analyses and long-term evolutionary studies because its loci are located in well-conserved housekeeping regions distributed across the chromosome (Fig. 1) [17,19]. This provides greater stability and coherence for defining long-term population structure. In contrast, the Oxford scheme suffers from several technical and biological limitations [19,20]. Some of its loci are positioned in close proximity within only one half of the chromosome (Fig. 1), potentially reducing the independence of allelic variation, leaving much of the chromosome unsampled and limiting phylogenetic resolution [20]. A serious issue was identified in the Oxford MLST scheme when the ST derived from whole-genome assemblies did not match the ST obtained using the original PCR primers. This discrepancy arose because the primer-binding sites for the *gpi* and *cpn60* genes were inadvertently included in the regions used for allele calling, generating artefactual alleles and giving rise to STs (e.g. ST92 and ST109) that do not correspond to real biological types [20]. At the time, the *gpi* locus was also noted as unusually variable [21], but it was not yet understood that *gpi* was located with the capsule biosynthesis region (KL), which is one of the most recombinogenic and horizontally exchanged regions in the *A. baumannii* genome [1,22]. This genomic context explains the allelic diversity originally attributed to *gpi* and highlights why loci positioned within or near the KL region are inherently unsuitable for stable MLST classification. For these and other reasons [19], the Pasteur MLST scheme, which avoids loci associated with the KL region, provides a more stable and phylogenetically coherent framework for defining clonal boundaries and interpreting long-term population structure.

**Fig. 2.**
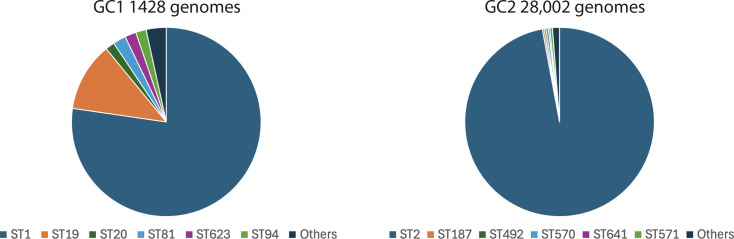
Proportion of genomes assigned to founder STs (ST1 for GC1 and ST2 for GC2) versus SLVs and DLVs.

Under the Pasteur scheme, GC1 is centred on ST1, while GC2 is centred on ST2 [17]. Numerous studies and reviews have documented the epidemiological significance of these clones, linking them to the spread of major resistance islands such as AbaR in GC1 and AbGRI1–AbGRI4 in GC2 [1,6, 23, 24]. It is widely recognized that GC1 and GC2 each encompass multiple related STs, namely single-locus variants (SLVs) or double-locus variants (DLVs) of ST1 or ST2 [1,17, 25, 26].

As new STs are continually deposited in the PubMLST database (https://pubmlst.org [27]), the possible boundaries of GC1 and GC2 have expanded considerably beyond the handful of types initially described (ST1: ST7, ST8, ST19 and ST20; and ST2: ST45 and ST47) [17]. This expansion creates challenges for comparative genomics, surveillance and researchers seeking to link phenotypic or epidemiological traits to clone assignment. Although earlier MLST [1,17, 19, 25, 28] analyses provide useful partial overviews or compilations of small numbers of SLVs and DLVs, there is currently no single, systematic resource that enumerates all STs belonging to GC1 and GC2. With the increasing reliance on whole-genome sequencing for surveillance, and multiple publications using STs to compile counts of genomes within a clone [8,10], consistent and transparent definitions of these clones are essential for accurate classification and comparative analysis. However, published studies frequently refer to clones by their founder ST alone without acknowledging the full breadth of related STs within each complex [9]. In some cases, isolates belonging to a CC are described simply by their observed ST, while in others, STs that clearly fall within an established CC are not recognized as such; for example, ST19 is not widely recognized as GC1 [13,14], leading to inconsistencies across the literature. These omissions complicate comparative genomics and hinder comparison of epidemiological findings across studies.

Here, this gap is addressed by developing a custom Python pipeline and systematically identifying all STs in the Pasteur MLST scheme that fall within GC1 or GC2, defined as SLVs and DLVs of ST1 or ST2 and their subsequent derivatives. Other predominant clones, including ST25, ST79, ST78, ST85 and ST499, are also examined. This analysis provides an updated catalogue of STs that constitute the major global clones and offers a framework for consistent classification in future genomic epidemiology studies.

## Methods

### Dataset and identification of global clone members

All *A. baumannii* MLST allele sequences and ST definitions from the Pasteur scheme [17] were downloaded from the PubMLST database (https://pubmlst.org/abaumannii). The dataset included all allelic profiles available on 24 July 2025. To determine global clone membership, a custom Python script (ab_pasteur_slv_dlv.py) was developed to identify all SLVs and DLVs (or TLVs if required) of a specific query ST. The script imports the PubMLST allelic profile data and uses the Pandas library (v2.3.2) to perform pairwise comparisons across STs, identifying those differing by one or two loci from the query ST (e.g. ST1 for GC1 and ST2 for GC2).

### Genome dataset analysis

To evaluate the impact of broadened clone definitions, all publicly available *A. baumannii* genomes were analysed. A total of 41,951 genome assemblies (including 990 complete genomes) were downloaded from GenBank on 16 July 2025 by querying the NCBI FTP site (https://ftp.ncbi.nlm.nih.gov/). The downloaded genome assemblies were typed using the updated (January 2026) MLST package (version 2.32.2, https://github.com/tseemann/mlst) to assign STs in the *A. baumannii* Pasteur scheme. Assemblies were included in downstream analyses only if a valid ST could be assigned using the Institut Pasteur MLST scheme. Assemblies that failed MLST allele calling or produced incomplete MLST profiles were excluded. The number of genomes classified as GC1 or GC2 under two criteria was compared, first including only ST1 or ST2 and second incorporating all SLVs and DLVs of these founder STs. This analysis was carried out in a Jupyter notebook, using Pandas (v2.3.2) to parse the MLST results and calculate genome counts.

### Allele co-variation analysis

To investigate whether MLST loci tend to vary together, an allele co-variation analysis was performed using the *A. baumannii* Pasteur scheme data. The datasets of SLVs and DLVs of ST1 and ST2 were used as input, containing the allele numbers for each of the seven Pasteur loci (*cpn60*, *fusA*, *gltA*, *pyrG*, *recA*, *rplB* and *rpoB*). For each compared ST, loci were coded as either identical to or different from the ST founder allele. Pairwise associations between loci were then quantified using a Phi coefficient to measure the strength of co-variation in allele changes. Statistical independence was assessed for each locus pair using χ² tests, with Bonferroni correction for multiple comparisons. Correlations and significance results were visualized as a heatmap of Phi coefficients. All analyses were conducted in Python (v3.12) using Pandas (v2.3.2), scipy (v1.13), statsmodels (v0.14) and seaborn (v0.13).

### Data availability

The Python scripts for ST comparison and genome dataset analysis are available on GitHub at https://github.com/charmer-git/ST. The up-to-date GC1 and GC2 ST lists are also available at the same GitHub repository.

## Results

### Overview

A custom Python workflow was developed (https://github.com/charmer-git/ST) to systematically identify all SLVs and DLVs associated with a given founder ST in any MLST scheme, e.g. those in PubMLST. The tool was used to interrogate the PubMLST database (last searched 24 July 2025) to identify SLVs/DLVs of *A. baumannii* ST1, ST2, ST25, ST78, ST79 and ST85 using the Pasteur MLST scheme. These are listed in Tables 1, 2 and S1–S6, available in the online Supplementary Material.

**Table 1.** SLVs of ST1 (GC1)

	Pasteur MLST loci							
ST	*cpn60*	*fusA*	*gltA*	*pyrG*	*recA*	*rplB*	*rpoB*	Genome count
**1**	**1**	**1**	**1**	**1**	**5**	**1**	**1**	**1083**
7	1	1	1	2	5	1	1	0
8	1	1	1	1	1	1	1	0
19	1	2	1	1	5	1	1	165
20	3	1	1	1	5	1	1	22
81	1	1	1	1	5	1	2	31
116	1	1	1	4	5	1	1	0
125	2	1	1	1	5	1	1	1
160	1	1	1	1	5	1	41	3
202	1	4	1	1	5	1	1	0
230	1	6	1	1	5	1	1	0
231	1	1	1	1	5	1	4	1
233	1	1	8	1	5	1	1	0
310	1	54	1	1	5	1	1	0
315	1	56	1	1	5	1	1	4
408	1	1	1	1	7	1	1	0
409	1	1	1	1	2	1	1	1
460	1	1	1	1	5	1	64	1
623	1	1	2	1	5	1	1	27
642	1	1	1	1	9	1	1	0
717	1	1	1	1	5	1	30	4
734	1	1	1	1	3	1	1	21
736	1	3	1	1	5	1	1	0
826	1	1	1	1	5	8	1	0
881	1	1	1	1	5	1	130	2
902	1	1	1	1	13	1	1	0
983	1	1	1	1	5	1	5	0
986	1	1	1	1	5	4	1	0
988	46	1	1	1	5	1	1	0
1090	1	151	1	1	5	1	1	16
1192	1	167	1	1	5	1	1	0
1208	1	168	1	1	5	1	1	0
1214	1	1	1	1	5	95	1	0
1475	1	1	1	1	5	123	1	1
1521	1	39	1	1	5	1	1	0
2026	1	1	14	1	5	1	1	0
2595	1	229	1	1	5	1	1	1
2806	1	1	1	1	248	1	1	0
2841	1	1	1	1	5	1	574	1

**Table 2.** DLVs of ST1 (GC1)

	Pasteur MLST loci							
ST	*cpn60*	*fusA*	*gltA*	*pyrG*	*recA*	*rplB*	*rpoB*	Genome count
**1**	**1**	**1**	**1**	**1**	**5**	**1**	**1**	**1083**
94^*^	1	2	2	1	5	1	1	25
101	1	1	1	2	1	1	1	0
173	26	4	1	1	5	1	1	0
174^*^	1	2	1	1	5	1	5	0
194	3	1	15	1	5	1	1	0
328^†^	1	1	1	25	5	1	2	9
493^*^	1	2	1	1	5	1	2	0
589	1	1	2	1	9	1	1	0
592^*^	2	2	1	1	5	1	1	1
637	1	1	10	1	6	1	1	0
646^†^	1	1	96	1	5	1	2	0
718^*^	1	2	1	1	5	1	30	0
829	1	56	124	1	5	1	1	0
980^†^	69	1	1	1	5	1	2	0
1106	2	1	2	1	5	1	1	5
1217	3	3	1	1	5	1	1	0
1219	3	1	1	1	7	1	1	0
1221	3	1	1	4	5	1	1	0
1246	5	1	1	1	9	1	1	0
1387	3	1	1	1	5	1	41	0
1439	3	1	1	1	5	4	1	0
1517^*^	1	2	1	1	5	2	1	0
1853	1	1	1	2	3	1	1	3
2137	344	1	1	115	5	1	1	0
2521^*^	1	2	1	265	5	1	1	0

*SLV of ST19.

†SLV of ST81.

A total of 41,951 genome assemblies annotated as *A. baumannii* were downloaded from GenBank and typed using the Pasteur MLST scheme with the MLST package (https://github.com/tseemann/mlst) available in December 2025 (version 2.25.0). Valid STs were assigned to 38,598 genomes (92%). However, we noticed that ST1090, which was recently detected in a large collection of GC1 genomes [29], was not detected. To explore this further, a representative ST1090 genome was tested both using the MLST package and directly in PubMLST, and an ST was returned only by PubMLST. Hence, other STs may also be missing due to previously undetected failures associated with the existing MLST calling pipeline. As the failure to detect ST1090 could also be because the MLST database used in our searches was out of date, we checked for updates and reran the MLST calling on the 41,951 genomes using the MLST release updated in January 2026 (version 2.32.2), which was not available when the analysis was first performed. This assigned STs to a further 2,208 genomes, leaving only 1,145 genomes (2.7%) without a valid ST. The latter group was also examined further (see below). All subsequent analyses in this manuscript were performed using the January 2026 version of MLST.

### Identification of GC1-associated STs

Applying the tool to ST1, the founder of GC1, revealed that GC1 encompasses far more STs than are captured by the founder ST alone. Across the PubMLST database, a total of 64 STs were identified as belonging to GC1, comprising ST1, 38 SLVs (Table 1) and 25 DLVs (Table 2). Several ST1 DLVs were SLVs of either ST19 or ST81 (marked in Table 2) consistent with the independent trajectory of the ST1, ST19 and ST81 lineages [1,25, 26]. The *fusA* locus showed the highest level of allelic diversity among GC1-associated STs, with 12 distinct alleles detected.

To assess how reliance on ST1 alone may affect detection of GC1-related genomes, the expanded set of GC1-associated STs was applied to 40,806 publicly available *A. baumannii* genome assemblies for which ST had been determined using the updated MLST package (https://github.com/tseemann/mlst). Using ST1 alone, 1,083 genomes were detected. When all identified SLVs and DLVs were included, an additional 345 genomes were incorporated (ranging from 1 to 165 per SLV/DLV), giving a total of 1,428 genomes assigned to GC1. This demonstrates that a sizeable proportion of GC1 diversity is missed if recognition of clone membership is confined solely to the founder ST (Fig. 2). The majority of the additional GC1 genomes are ST19 (*n*=165), ST94 (*n*=25, an SLV of ST19), ST81 (*n*=31) and ST623 (*n*=27, an SLV of ST81). The additional genomes account for 24.2% of all GC1-classified isolates, and the complete GC1 collection represents 3.4% of all sequenced *A. baumannii* isolates, up from 2.6% when only ST1 is included.

The additional 345 genomes were distributed across only 22 of the 64 GC1-associated SLVs and DLVs identified from PubMLST. Hence, most of the STs predicted to belong to GC1 were not detected among publicly available genome assemblies. This includes several early STs, associated with GC1, including ST7 and ST8 [17], that were not observed in any sequenced genomes in the GenBank dataset. This may reflect allele profiles that have been submitted to the MLST database for which corresponding genome assemblies have not yet been generated or released. Alternatively, it may represent erroneous or artefactual allele combinations.

We also examined the concordance between the STs detected here and in published GC1 phylogenies [1,25, 26, 29] and found that they included ST1 plus 9 of the 22 STs for which genomes were detected here, and no further cases of STs that were not detected here.

### Identification of GC2-associated STs

The same workflow was applied to ST2, the founder ST of GC2. This identified 164 GC2 STs, ST2 and 115 SLVs and 48 DLVs of ST2 in PubMLST (Tables 3 and S1). Within this group, the *rpoB* locus had the greatest allelic variation, with 32 different alleles observed among GC2-associated STs.

**Table 3.** Number of SLVs and DLVs of representative dominant STs

Pasteur ST	SLVs	DLVs
ST1	38	25
ST2	115	48
ST25	26	16
ST78	6	3
ST79	20	18
ST85	3	6
ST499	6	4

Searching the genome dataset with ST2 alone assigned 26,962 genomes to GC2. Inclusion of all ST2 SLVs and DLVs added 1,040 further genomes (ranging from 1 to 124 genomes per SLV/DLV), producing a total of 28,002 GC2-assigned genomes. The most prevalent additional STs include ST185 (*n*=89), ST570 (*n*=114) and ST571 (*n*=124, an SLV of ST570). The additional genomes account for 3.7% of all GC2-classified isolates, a significantly smaller proportion than for GC1. The 28,002 GC2 isolates represent 66.7% of all sequenced *A. baumannii* isolates, up from 64.2% when only ST2 was included.

The additional GC2 genomes were distributed across only 65 of the 164 additional SLVs and DLVs identified from the PubMLST database. Hence, like GC1, most of the predicted GC2 STs are not represented among publicly available genome assemblies.

### Application to other predominant STs

To assess whether the same SLV and DLV approach could delineate broader clonal frameworks beyond the two major global clones, the method was applied to five other clinically important *A. baumannii* STs: ST25, ST78, ST79, ST85 and ST499. Each of these clones is known to occur among multidrug-resistant isolates and to have been implicated in regional outbreaks or hospital persistence.

The analysis revealed that CC25 forms an extensive CC comprising ST25, 26 SLVs and 16 DLVs (Tables 3 and S2), consistent with its recognition as a long-established clone associated with international spread [11,12]. However, among 706 total CC25 genomes, only 530 were ST25, and only 8 of the ST25 SLVs (1 to 11 genomes) and 12 DLVs (1 to 83 genomes) were represented.

CC79 also showed substantial diversity, with a CC containing 20 SLVs and 18 DLVs (Tables 3 and S3). Again, among the 490 total CC79 genomes, only 286 were ST79, and only a small proportion of the SLVs (8 out of 20, with 1 to 107 genomes each) and DLVs (8 out of 18, with 1 to 12 genomes each) were found in the genome dataset.

In contrast, CC499, CC78 and CC85 exhibited more restricted structures consistent with more confined geographic spread, with fewer than seven SLVs/DLVs each (Tables 3 and S4–S6).

### Allele co-variation among MLST loci

Given the extensive recombination known to occur in *A. baumannii*, it was important to assess whether large recombination events might replace multiple MLST loci simultaneously, potentially causing coordinated allele changes. To test this, pairwise associations between alleles at the Pasteur MLST loci were evaluated using the Phi correlation coefficient for both GC1 (data not shown) and GC2 STs (Table 4). Across all locus pairs, correlations were uniformly weak (|Φ|≤0.08), and none remained statistically significant after correction for multiple testing (*P*>0.05). Even the two loci located closest together in the *A. baumannii* chromosome, *gltA* and *cpn60* (Fig. 1), which would be the most likely to co-vary under large-scale recombination, showed only negligible association (Φ=0.03). The loci with the stronger, albeit still very weak, association (Φ=0.08), *cpn60* and *fusA*, are located almost symmetrically opposite to one another in the *A. baumannii* chromosome. These findings indicate minimal co-variation among the Pasteur MLST loci, and the lack of detectable linkage supports the conclusion that these loci typically evolve independently through point mutation or localized recombination rather than through wholesale co-replacement in large recombination events.

**Table 4.** Correlation of GC2 loci^*^

Locus 1	Locus 2	Phi	*P*-value	Significant
*cpn60*	*fusA*	0.076864	0.736610	No
*fusA*	*recA*	0.076864	0.736610	No
*fusA*	*rplB*	0.065907	0.898867	No
*fusA*	*pyrG*	0.048619	1.000000	No
*cpn60*	*gltA*	0.027969	0.926287	No
*cpn60*	*recA*	0.004154	1.000000	No
*gltA*	*pyrG*	−0.023504	1.000000	No
*fusA*	*gltA*	−0.032892	1.000000	No
*pyrG*	*rpoB*	−0.042366	0.819454	No
*recA*	*rplB*	−0.069018	0.548818	No

*Only the top 10 positive and negative associations are shown.

### Assemblies without complete MLST profiles

Of the 41,951 genome assemblies examined, 1,145 (2.7%) did not yield a complete Pasteur MLST profile. Examination of these assemblies indicated several causes. In 888 cases, the allele combinations corresponded to novel STs not yet defined in the PubMLST database. Others (119 genomes) contained multiple partial copies or copies of one or more loci, preventing unambiguous allele assignment. This could be due to contamination or duplications and was not investigated further. Only 138 genomes lacked one or more MLST loci, consistent with fragmented assemblies or a chromosomal deletion. Importantly, all assemblies contained at least five of the seven Pasteur MLST loci characteristic of *A. baumannii*, supporting their taxonomic consistency with the species.

## Discussion

This study highlights the ongoing challenge of identifying CC members in *A. baumannii* using MLST. Although GC1 and GC2 are often assumed to consist solely of ST1 and ST2, respectively, this analysis demonstrates that reliance on these founder STs alone substantially underestimates the genomic diversity contained within the important CCs. Including SLVs and DLVs in the CC analysis revealed a considerably larger set of related STs, reflecting the complex evolutionary history and ongoing diversification of globally dominant clones. Despite increasing adoption of whole-genome phylogenies and core-genome MLST approaches, retention of traditional STs remains critically important, and the Pasteur MLST framework provides a long-established common language that connects contemporary genomic analyses to over three decades of epidemiological literature. Many foundational studies of *A. baumannii* resistance evolution, outbreak dynamics and lineage dissemination are defined in terms of STs [1,3, 17, 19, 30], and maintaining these identifiers ensures that new genomic findings remain interpretable within this historical context. In practical terms, reliance on founder STs alone would lead to systematic underestimation of the prevalence of dominant *A. baumannii* clones, with implications for antimicrobial resistance surveillance and for recognizing ongoing transmission or local diversification within healthcare settings.

The contrasting diversity profiles of GC1 and GC2 underscore their distinct evolutionary and epidemiological trajectories. The broader ST diversity within GC2 likely reflects both its successful evolutionary trajectory in hospital environments and the extensive sampling of this clone in surveillance studies worldwide, particularly those with a focus on carbapenem resistance. By comparison, the smaller but still substantial diversity observed in GC1 highlights that it, too, is a long-established clone of clinical importance [1], albeit one with a narrower footprint in current genomic datasets. Apparent differences in diversity may also partly reflect a resistance-associated sampling bias, as new STs frequently enter genomic databases only after acquiring antimicrobial resistance. Consequently, CCs where resistance has emerged more recently may appear less diverse simply because they have not yet been extensively sequenced.

A recent study proposed the existence of a single ‘epidemic super-lineage’ (ESL) encompassing the majority of *A. baumannii* genomes worldwide, including both GC1 and GC2 within a single, unified clade [31]. However, the data presented here support the continued recognition of GC1 and GC2 as distinct long-established epidemic CCs with separate allelic profiles, networks of STs and evolutionary trajectories. Their genomic differentiation is supported not only by MLST relationships but also by well-characterized clone-specific features, including the AbGRI resistance islands [32,34] that are unique to GC2 and the AbaR islands characteristic of lineage 1 of GC1 [23]. Collapsing these CCs into a single ESL obscures the independent emergence, spread and resistance evolution of two of the most important clones in modern hospital epidemiology. Adoption of the ESL concept would risk erasing decades of detailed clone-specific research and undermine the interpretability of existing genomic and clinical data.

Allelic differences detected by MLST can arise from a few single-nucleotide substitutions or from recombination events, which have long been recognized as major drivers of diversity in *A. baumannii* [1,22, 25, 35]. Such events can result in wholesale allele replacements that alter MLST-defined relationships, particularly around highly recombinant regions such as the capsule biosynthesis locus [21,22], while large chromosomal recombinations occurring outside the seven Pasteur MLST loci may remain undetected due to the dispersed locations of these markers. Despite these genome dynamics, the characteristic antibiotic resistance islands (AbGRI1, AbGRI2 and AbGRI3) remain reliable genomic markers for identifying GC2, even as the clone continues to diversify and accumulate additional locus variants.

Application of this analytical framework to other successful clones, including ST25, ST78, ST79, ST85 and ST499, confirmed its broad utility. The extensive SLV/DLV networks observed for ST25 and ST79 indicate long-term diversification and dissemination, while the smaller complexes surrounding ST78, ST85 and ST499 correspond to more regionally constrained or less studied CCs. These comparisons demonstrate that the SLV/DLV-based approach provides a consistent and transparent method to delineate CCs across *A. baumannii*, enabling direct comparison between long-established and emerging epidemic clones.

Only a subset of SLV/DLV-defined STs were represented among available genome assemblies. Whether this represents incomplete coverage of existing types among the assembled genomes in the publicly available databases, particularly for recently reported STs, or artefacts arising from earlier PCR-based MLST datasets remains to be determined. Integrating MLST data with whole-genome analyses will be essential to ensure consistency of CC detection between allele-based and genome-wide frameworks. The reproducible workflow described here can be regularly updated as PubMLST expands, providing a harmonized foundation for clonal classification and enhancing genomic surveillance of this globally significant pathogen. This workflow can also be applied to other important pathogens with complex clonal relationships, including *Klebsiella pneumoniae* [36,38] and *Pseudomonas aeruginosa* [39].

## Supplementary material

10.1099/mgen.0.001772 Supplementary Material 1.

## Abbreviations

CCs: clonal complexes
DLVs: double-locus variants
ESL: epidemic super-lineage
GC1: Global Clone 1
GC2: Global Clone 2
MAR: multiply antibiotic-resistant
SLVs: single-locus variants
ST1: sequence type 1
ST2: sequence type 2
STs: sequence types
TLVs: triple-locus variants
