# Supplementary material for: Comprehensive identification of sequence types belonging to Acinetobacter baumannii clonal complexes
Source: Microb Genom. 2026 Jul 1;12(7):001772. doi: 10.1099/mgen.0.001772 (PMC13322331; doi:10.1099/mgen.0.001772)
Supplement: Supplementary Material 1. [file mgen-12-01772-s001.pdf]

Table S1. GC2 SLVs and DLVs

	Pasteur MLST loci							
ST	*cpn60*	*fusA*	*gltA*	*pyrG*	*recA*	*rplB*	*rpoB*	Genome count
**2**	**2**	**2**	**2**	**2**	**2**	**2**	**2**	**26962**
SLVs								
45	2	6	2	2	2	2	2	16
47	2	13	2	2	2	2	2	3
97	2	2	36	2	2	2	2	0
98	1	2	2	2	2	2	2	6
104	2	2	2	2	2	2	14	4
115	2	2	2	1	2	2	2	0
143	2	2	2	2	2	2	38	0
183	2	23	2	2	2	2	2	0
184	43	2	2	2	2	2	2	0
185	2	2	2	2	2	2	42	4
187	2	2	2	2	2	2	43	89
195	2	2	2	2	2	1	2	33
247	2	2	2	2	2	31	2	0
254	2	2	2	25	2	2	2	1
257	2	9	2	2	2	2	2	0
261	2	2	2	2	2	2	29	0
263	2	4	2	2	2	2	2	0
380	2	2	2	2	4	2	2	1
389	2	64	2	2	2	2	2	0
391	2	2	58	2	2	2	2	0
393	2	2	2	31	2	2	2	0
414	2	2	2	2	2	37	2	5
415	2	2	2	2	68	2	2	16
487	2	2	1	2	2	2	2	0
489	2	2	2	2	2	8	2	0
492	2	3	2	2	2	2	2	81
523	2	2	2	2	99	2	2	1
524	2	97	2	2	2	2	2	1
525	2	2	2	2	2	50	2	1
526	2	2	2	2	100	2	2	1
570	2	2	2	2	2	2	4	114
576	2	2	2	2	2	2	92	0
580	2	2	2	2	2	52	2	0
597	2	2	2	2	7	2	2	0
600	2	2	2	2	2	2	1	55
604	2	2	2	55	2	2	2	38
632	2	2	2	2	2	2	96	65

633	2	101	2	2	2	2	2	0
641	2	1	2	2	2	2	2	69
645	2	103	2	2	2	2	2	0
661	2	106	2	2	2	2	2	1
662	2	2	2	58	2	2	2	1
663	2	2	8	2	2	2	2	0
664	2	2	2	2	2	4	2	8
668	2	2	100	2	2	2	2	1
670	2	2	102	2	2	2	2	1
671	2	2	2	2	113	2	2	1
672	2	107	2	2	2	2	2	0
674	2	2	2	2	114	2	2	1
678	2	2	2	2	22	2	2	0
697	3	2	2	2	2	2	2	1
699	103	2	2	2	2	2	2	0
713	2	2	2	2	2	2	52	0
724	2	2	2	2	2	2	5	15
745	2	2	2	2	2	2	101	19
823	2	125	2	2	2	2	2	2
880	2	2	2	2	2	77	2	1
922	2	2	2	75	2	2	2	6
996	2	2	2	2	2	2	49	0
997	2	2	2	2	2	2	44	0
998	2	2	2	2	2	2	20	0
999	2	2	2	2	2	2	97	0
1003	2	2	2	2	2	2	51	0
1004	2	2	2	2	2	2	63	0
1009	2	2	2	2	2	2	3	0
1067	161	2	2	2	2	2	2	0
1078	2	146	2	2	2	2	2	0
1108	2	156	2	2	2	2	2	1
1109	2	2	153	2	2	2	2	3
1154	2	2	2	2	2	2	148	0
1160	2	161	2	2	2	2	2	42
1202	4	2	2	2	2	2	2	0
1253	2	2	2	2	2	2	162	0
1313	2	2	182	2	2	2	2	0
1449	2	2	2	2	67	2	2	0
1474	2	2	2	2	2	2	201	0
1477	2	2	7	2	2	2	2	0
1532	2	2	2	114	2	2	2	0
1533	2	2	2	115	2	2	2	0
1537	2	2	222	2	2	2	2	2

1544	2	2	2	2	2	129	2	1
1550	2	226	2	2	2	2	2	0
1552	27	2	2	2	2	2	2	0
1553	2	225	2	2	2	2	2	0
1555	2	2	181	2	2	2	2	19
1573	2	228	2	2	2	2	2	3
1575	2	2	2	2	2	2	217	0
1579	2	229	2	2	2	2	2	77
1580	2	230	2	2	2	2	2	34
1644	288	2	2	2	2	2	2	2
1645	2	245	2	2	2	2	2	1
1723	290	2	2	2	2	2	2	0
1851	2	2	2	2	2	2	317	1
2125	2	2	2	2	248	2	2	0
2128	2	2	2	2	242	2	2	0
2131	2	2	2	2	2	86	2	0
2249	2	2	2	2	2	264	2	0
2485	2	2	2	2	2	16	2	1
2513	2	2	2	2	8	2	2	1
2523	2	443	2	2	2	2	2	5
2528	2	444	2	2	2	2	2	0
2529	2	445	2	2	2	2	2	0
2533	2	2	2	2	503	2	2	0
2562	2	148	2	2	2	2	2	2
2564	2	2	68	2	2	2	2	2
2571	2	2	2	2	2	3	2	1
2581	2	2	2	2	550	2	2	0
2614	2	2	471	2	2	2	2	0
2709	2	2	2	2	2	2	550	0
2711	2	514	2	2	2	2	2	2
2716	2	2	2	2	615	2	2	1
2724	509	2	2	2	2	2	2	0
2736	601	2	2	2	2	2	2	0
2745	2	2	2	2	620	2	2	0
2866	2	534	2	2	2	2	2	0
DLVs								
96	1	2	36	2	2	2	2	0
129	3	2	3	2	2	2	2	18
196	2	2	13	2	2	1	2	0
344	2	2	1	1	2	2	2	0
379	3	2	2	2	2	1	2	0
518	5	2	2	2	2	2	5	0
571	2	2	2	2	104	2	4	124

577	27	2	7	2	2	2	2	7
579	2	3	2	2	2	4	2	0
593	2	2	2	4	2	2	4	0
596	1	1	2	2	2	2	2	0
603	2	2	93	2	2	2	94	0
673	103	2	103	2	2	2	2	1
684	27	4	2	2	2	2	2	0
700	2	2	2	2	100	2	1	0
703	103	2	2	2	68	2	2	0
706	2	2	2	6	2	2	1	0
710	103	2	2	2	100	2	2	0
712	103	2	2	2	2	2	4	0
716	103	2	2	2	2	2	1	0
990	3	2	2	2	3	2	2	1
1001	2	2	2	2	3	1	2	0
1005	3	2	2	2	2	2	5	0
1103	164	2	2	2	5	2	2	0
1107	2	148	2	2	2	1	2	0
1143	6	2	8	2	2	2	2	10
1204	103	2	2	59	2	2	2	0
1236	2	2	2	53	2	2	99	0
1239	2	156	98	2	2	2	2	0
1240	2	2	2	2	2	50	148	0
1244	2	2	23	55	2	2	2	0
1245	2	2	2	2	114	2	96	0
1312	2	6	181	2	2	2	2	0
1516	2	2	2	2	2	1	1	0
1538	2	2	222	2	2	86	2	0
1559	2	3	2	2	2	2	4	0
1572	2	2	2	2	5	2	1	1
1828	3	2	2	2	7	2	2	9
2126	2	2	2	2	248	86	2	0
2130	2	2	2	2	68	57	2	0
2151	2	2	414	2	2	2	422	0
2152	2	2	415	2	461	2	2	0
2262	2	6	2	2	2	2	451	0
2478	5	2	4	2	2	2	2	3
2544	2	2	2	2	2	1	137	1
2549	2	6	2	2	2	2	4	2
2721	5	2	2	2	5	2	2	0
2753	2	2	2	2	104	2	30	0

Table S2. ST25 SLVs and DLVs.

ST	Pasteur MLST loci							Genome count
	cpn60	*fusA*	*gltA*	*pyrG*	*recA*	*rplB*	*rpoB*	
**25**	**3**	**3**	**2**	**4**	**7**	**2**	**4**	**530**
SLVs								
228	40	3	2	4	7	2	4	1
300	3	3	2	4	3	2	4	0
304	3	3	2	4	29	2	4	0
307	3	3	2	4	7	2	30	5
402	3	3	2	1	7	2	4	1
491	3	3	2	4	2	2	4	0
619	3	3	2	4	7	2	2	11
627	3	6	2	4	7	2	4	0
630	3	3	2	4	108	2	4	0
732	3	35	2	4	7	2	4	0
945	3	3	2	4	7	4	4	0
991	3	3	2	4	7	2	5	0
1065	3	3	2	4	7	1	4	0
1080	3	147	2	4	7	2	4	0
1118	3	3	150	4	7	2	4	0
1124	3	3	2	4	7	2	147	1
1125	3	3	2	4	7	89	4	0
1193	3	3	2	4	7	2	155	0
1218	3	3	2	4	15	2	4	0
1448	3	3	2	4	7	114	4	2
1576	3	3	2	4	244	2	4	0
1581	3	3	13	4	7	2	4	0
1595	3	3	2	4	247	2	4	0
1631	3	3	2	4	115	2	4	0
2159	3	407	2	4	7	2	4	1
2613	3	3	2	277	7	2	4	0
DLVs								
110	3	2	2	4	7	2	2	0
113	3	3	3	4	7	4	4	83
240	3	3	2	5	7	2	51	30
243	3	3	2	2	9	2	4	17
324	3	3	2	4	2	2	2	0
588	3	3	2	2	7	2	2	0
612	3	3	3	4	7	9	4	0
690	3	3	2	1	7	2	14	16
760	3	1	2	1	7	2	4	2
1007	3	3	2	5	7	2	44	0

1085	3	3	2	1	7	4	4	0
1459	3	4	2	2	7	2	4	2
1625	3	2	2	2	7	2	4	1
1850	3	3	7	1	7	2	4	0
1902	3	317	2	4	360	2	4	2
2526	6	3	8	4	7	2	4	2

Table S3. ST79 SLVs and DLVs

	Pasteur MLST loci							
ST	*cpn60*	*fusA*	*gltA*	*pyrG*	*recA*	*rplB*	*rpoB*	Genome count
**79**	**26**	**2**	**2**	**2**	**29**	**4**	**5**	286
SLVs								
156	26	2	2	2	29	4	4	107
169	26	2	2	2	29	1	5	0
170	26	4	2	2	29	4	5	0
171	26	16	2	2	29	4	5	0
175	26	1	2	2	29	4	5	0
259	26	2	2	2	2	4	5	0
298	1	2	2	2	29	4	5	1
422	26	72	2	2	29	4	5	26
730	26	6	2	2	29	4	5	7
836	26	2	2	1	29	4	5	0
885	26	2	2	2	29	5	5	0
903	26	2	2	2	29	4	1	0
957	26	2	2	2	29	53	5	0
958	6	2	2	2	29	4	5	0
1163	26	2	2	2	29	4	2	4
1196	26	2	2	2	29	4	29	1
1325	26	3	2	2	29	4	5	0
1466	26	199	2	2	29	4	5	0
2248	26	2	436	2	29	4	5	15
2565	2	2	2	2	29	4	5	6
DLVs								
141	26	2	2	1	43	4	5	12
163	3	2	2	2	2	4	5	1
167	26	2	2	2	9	1	5	0
190	1	2	2	2	11	4	5	1
363	1	2	2	2	15	4	5	0
765	3	2	2	2	6	4	5	2
874	6	115	2	2	29	4	5	0
966	3	2	2	2	44	4	5	1
985	26	2	2	2	29	25	2	0
1211	1	2	2	2	73	4	5	1
1584	3	2	2	2	102	4	5	0
1878	6	1	2	2	29	4	5	8
1889	8	2	2	2	17	4	5	0
2190	486	1	2	2	29	4	5	0
2238	3	2	2	2	5	4	5	11

2465	26	6	2	2	29	1	5	0
2599	534	3	2	2	29	4	5	0
2796	620	2	2	2	29	4	4	0

Table S4. ST78 SLVs and DLVs

	Pasteur MLST loci							
ST	*cpn60*	*fusA*	*gltA*	*pyrG*	*recA*	*rplB*	*rpoB*	Genome count
**78**	**25**	**3**	**6**	**2**	**28**	**1**	**29**	**295**
SLVs								
676	25	109	6	2	28	1	29	0
1077	25	3	6	2	28	1	2	18
1344	25	3	6	2	28	1	4	1
1558	25	3	6	2	28	1	1	0
1907	25	3	6	2	28	1	328	2
2826	25	3	6	2	2	1	29	2
DLVs								
177	3	3	6	2	4	1	29	0
647	3	3	6	2	51	1	29	11
1424	25	3	6	2	5	1	1	0
2572	25	5	11	2	28	1	29	0

Table S5. ST85 SLVs and DLVs

	Pasteur MLST loci							
ST	*cpn60*	*fusA*	*gltA*	*pyrG*	*recA*	*rplB*	*rpoB*	Genome count
**85**	**5**	**2**	**4**	**1**	**3**	**3**	**4**	**128**
SLVs								
6	5	4	4	1	3	3	4	8
464	5	2	4	1	3	4	4	10
2559	5	2	2	1	3	3	4	1
DLVs								
528	5	98	4	1	3	4	4	1
708	5	2	4	1	30	3	2	0
889	5	2	4	1	30	4	4	0
919	5	4	4	1	3	3	14	0
972	5	2	4	1	3	1	3	2
2094	1	2	4	1	3	4	4	0

Table S6. ST499 SLVs and DLVs

			Pasteur MLST loci					
ST	*cpn60*	*fusA*	*gltA*	*pyrG*	*recA*	*rplB*	*rpoB*	Genome count
**499**	**5**	**2**	**39**	**2**	**3**	**1**	**5**	**2491**
SLVs								
123	5	2	1	2	3	1	5	0
135	5	2	39	2	39	1	5	0
192	5	35	39	2	3	1	5	0
1180	5	2	39	2	168	1	5	0
2139	5	2	39	2	3	1	2	123
2511	5	2	39	2	28	1	5	1
DLVs								
268	3	2	2	2	3	1	5	4
377	1	2	2	2	3	1	5	0
1074	1	2	39	2	39	1	5	2
2206	3	2	430	2	3	1	5	0
